# Liposomal Forms of Fluoroquinolones and Antifibrotics Decorated with Mannosylated Chitosan for Inhalation Drug Delivery

**DOI:** 10.3390/pharmaceutics15041101

**Published:** 2023-03-29

**Authors:** Irina Le-Deygen, Anastasia Safronova, Polina Mamaeva, Yana Khristidis, Ilya Kolmogorov, Anna Skuredina, Peter Timashev, Elena Kudryashova

**Affiliations:** 1 Chemistry Department, Lomonosov Moscow State University, Moscow 119991, Russia; 2 Institute for Regenerative Medicine, Sechenov First Moscow State Medical University (Sechenov University), Moscow 119991, Russia; 3 Department of Polymers and Composites, N.N. Semenov Federal Research Center for Chemical Physics, Moscow 119991, Russia

**Keywords:** liposomes, antibacterial drugs, inhaled drug delivery, antifibrotic drugs, spectroscopy, chitosan

## Abstract

The severe course of COVID-19 leads to the long-terming pulmonary diseases, such as bacterial pneumonia and post-COVID-19 pulmonary fibrosis. Thus, the essential task of biomedicine is a design of new effective drug formulations, including those for inhalation administration. In this work, we propose an approach to the creation of lipid–polymer delivery systems for fluoroquinolones and pirfenidone based on liposomes of various compositions decorated with mucoadhesive mannosylated chitosan. A generalizing study on the physicochemical patterns of the interactions of drugs with bilayers of various compositions was carried out, and the main binding sites were identified. The role of the polymer shell in the stabilization of vesicles and the delayed release of the contents has been demonstrated. For the liquid–polymer formulation of moxifloxacin, a prolonged accumulation of the drug in lung tissues was found after a single endotracheal administration to mice, significantly exceeding the control intravenous and endotracheal administration of the drug.

## 1. Introduction

To date, the pandemic of a new coronavirus infection has dramatically changed the concept of treating infections and forced countries to take unprecedented measures to protect the population. Millions of patients required intensive medical care and then long-term rehabilitation [1,2]. Often, COVID-19 is accompanied by a secondary bacterial infection—pneumonia, which is treated with antibacterial drugs such as fluoroquinolones [3]. The issue of the subsequent rehabilitation of patients after severe COVID-19 is very acute. It is known that the SARS-CoV-2 virus is able to trigger an immune reaction in the lungs, eventually leading to tissue fibrosis, similar to idiopathic pulmonary fibrosis [4]. Some researchers compare COVID-associated pulmonary fibrosis with the tsunami that is inevitable after the epidemic of a new coronavirus infection [5].

Optimism raises the possibility of using pirfenidone [6], currently approved by the FDA for the treatment of idiopathic pulmonary fibrosis.

Currently, intravenous or oral administration of both pirfenidone and fluoroquinolones is recommended; however, the bioavailability in lung tissues remains low, and side effects can significantly slow down the recovery. It is known that the use of inhaled forms of antibacterial drugs can be more effective in the treatment of pneumonia [7], including in a number of difficult cases when another form of therapy is impossible [8]. Indeed, inhaled administration can provide a higher effectiveness of therapy and less side effects for both antifibrotic drugs [9,10] and fluoroquinolones [11].

Thus, the development of new dosage forms for the treatment of severe forms of secondary pneumonia, as well as for the subsequent rehabilitation of patients with developed pulmonary fibrosis, is relevant.

To solve this problem, we propose to use the approach developed in our laboratory consisting of loading the drug into liquid–polymer nanoparticles [12,13]. Previously, we studied the effect of polymer properties on the structural and functional properties of liposomal forms of fluoroquinolones (moxifloxacin [14,15] and levofloxacin [16]) using PEGylated and mannosylated chitosan derivatives. We found the way to fine tailor liquid–polymer nanoparticles by means of variations of chitosan derivative nature, e.g., varying storage stability, controlled release, etc.

Recently several papers were published devoted to the inhaled liposomal form of some antituberculosis drugs such as rifampicin [17,18]. An in vivo test with small animals reported that almost 65% of the dose was achieved in the lungs. Moreover, the advantage of liquid–polymer particles lies in the versatility of this platform. Liposomes have been proven as an inhalation delivery system, including for the treatment of idiopathic pulmonary fibrosis [19]. It is known that lipid-based delivery systems are preferred for delivery to the lungs [20]. Thus, it was shown that, after inhalation, the accumulation of liposomes in the lungs after 24 h was significantly higher than that of quantum dots, dendrimers, and mesoporous silicon nanoparticles. By varying the composition and size of liposomes, it is possible to control the efficiency of liposome delivery to the lungs using a nebulizer [21]. For the delivery of fluoroquinolones, liposomes and liquid–polymer vesicles seem to be one of the best solutions [22] due to their high drug loading capacity, ease of preparation, and the ability to control physicochemical properties.

Thus, lipid-based delivery systems for fluoroquinolones and antifibrotic drugs have significant advantages, including high biocompatibility, high encapsulation efficiency, and variability of properties. On the other hand, the main drawback of lipid systems includes low stability.

To solve the problem of liposome stability and surface functionalization, many researchers [23,24,25,26] have proposed the formation of polymeric shells. However, the polymer here must meet a number of requirements, in addition to stabilization, namely effective interaction with the liposomal membrane, biocompatibility, absence of an immune response, the possibility of chemical modification, and covalent crosslinking with address labels. Among the studied polymers, there are both synthetic [27] and natural polymers [28,29]. For a long time, researchers have shown considerable interest in chitosan as a promising ligand for the stabilization of liposomes [30]. However, it is widely known that chitosan forms gels in neutral and alkaline media [31]. Therefore, in the last decade, chitosan derivatives have been used, for example, N-amino alkylchitosan [32], thiolchitosan [33], and copolymers of chitosan with polyacrylic acid [34]. Moreover, chitosan by itself is able to increase the efficacy of therapy, for example, metformin [35] and several vaccines’ cellular immunity [36]. The technology to produce PEGylated chitosan has been previously published [37]. PEGylation can significantly increase the solubility of chitosan. Thus, a polymer shell stabilizes liposomes and also provides the required physicochemical properties, depending on the type of substituent on the polymer, including giving them the effect of active targeting.

Attention is also drawn to the application of mannosylated chitosan for drug delivery to the lungs [38]. It is known that alveolar macrophages of the human lungs, often affected by various pathogens [39], express specific mannose-binding receptors on their surfaces [40,41]. Thus, the use of mannosylated chitosan with mucoadhesive properties seems promising for the creation of liquid–polymer systems for inhalation drug delivery.

Thus, the main goal of this study is to discover the structural and functional properties of liposomal forms of fluoroquinolones and pirfenidone decorated with mannosylated chitosan for inhalation administration.

## 2. Materials and Methods

### 2.1. Materials

Levofloxacin, moxifloxacin, pirfenidone, and acetonitrile—Sigma Aldrich, St. Louis, MO, USA; dipalmitoylphosphatidylcholine (DPPC), cholesterol (Chol), and 16:0 Cardiolipin (1′,3′-bis [1,2-dipalmitoyl-sn-glycero-3-phospho]-glycerol (sodium salt)) (CL)—Avanti Polar Lipids, Alabaster, AL, USA. Sodium phosphate buffer tablets for solution preparation were from Pan-Eco, Moscow, Russia. Mannosylated chitosan with M_w_ 6000 and degree of mannosylation 15% (4 mannoses per 1 chitosan chain) were synthesized and characterized according to the standard procedure described in [15].

### 2.2. Liposome Preparation

Thin film hydration followed by the ultrasonication technique were applied to prepare the liposomes. Briefly, the chloroform solution of lipids (DPPC, Chol, and CL, all 25 mg/mL) in desirable ratio was transferred to a round bottom flask, and the solvent was removed to a vacuum rotary evaporator at a temperature less than 55 °C. The resulting thin film was dispersed in 0.02 M sodium phosphate buffer, pH 7.4, according to the following procedure. Firstly, the flask with buffer solution was exposed to an ultrasonic bath (37 Hz) for 5 min; after that, the nontransparent suspension was transferred to the plastic tube and sonicated (22 kHz) for 600 s (2 × 300 s) in a continuous mode with constant cooling on a 4710 “Cole-Parmer Instrument” disperser.

### 2.3. Liposomal Form of Drugs Preparation

To obtain liposomal forms of moxifloxacin, levofloxacin, or pirfenidone, the same procedure was applied with the following changes: a thin film of lipids was dispersed with 0.02 M sodium phosphate-buffered solution, pH 7.4, containing drugs, namely pirfenidone (2 mg/mL), levofloxacin (4 mg/mL), or moxifloxacin (4 mg/mL). The unloaded drug was separated by dialysis against 0.02 M sodium phosphate-buffered solution, pH 7.4 (Serva, Heidelberg, Germany; MW cut-off 3500) for 120 min.

### 2.4. DLS Measurements

The hydrodynamic diameter Dh and ζ-potential of each liposomal system under consideration were measured using a Zetasizer Nano S Malvern (Malvern Panalytical Ltd., Malvern, UK) (4 mW He–Ne laser, 633 nm) in a thermostated cell at 22 °C. Autocorrelation curves were analyzed with Malvern software.

### 2.5. UV–Vis Spectroscopy

UV–Vis spectra were obtained from UV–Vis spectrometer AmerSharm Biosciences UltraSpec 2100 pro (Holliston, MA, USA) in a 1 mL quartz cuvette from Hellma Analytics (Müllheim, Germany). The main characteristic peaks of pirfenidone, levofloxacin, and moxifloxacin were observed at 310 nm, 287 nm, and 295 nm, correspondingly.

### 2.6. ATR-FTIR Spectroscopy

ATR-FTIR spectra were obtained from a Tensor 27 ATR-FTIR Fourier spectrometer (Bruker, Germany) equipped with a MCT detector cooled with liquid N_2_ and a thermostat (Huber, Raleigh, NC, USA). The measurements were carried out in a BioATR II thermostated cell (Bruker, Germany) using a single reflection ZnSe element at 22 °C and continuous purging of the system with dry air using a compressor (Redditch, UK). In a typical experiment, the volume of the sample was 50 µL, and the spectra were recorded three times in the range from 4000 to 950 cm^−1^ with a resolution of 1 cm^−1^, with 70 scans in each spectrum. The background spectra were obtained in the same way and were automatically subtracted by the software (Opus 7.0 Bruker). When recording the ATR-FTIR spectra of drug-loaded or polymer-coated vesicles, a solution of drug and polymer in equal concentration was used as a background solution. For phase transition studies, the temperature was varied by means of a thermostat in the range from 20 °C to 55 °C. For each temperature, the background spectra were recorded.

### 2.7. Encapsulation Efficacy and Drug–Lipids Mass Ratio Calculation

Encapsulation efficacy (EE) of pirfenidone (λ = 310 nm), levofloxacin (λ = 287 nm), and moxifloxacin (λ = 295 nm) were calculated with material balance, according to the formula:

$$Encapsulation\;efficacy=\frac{{\nu \left(Drug\right)}_{total}-{\nu \left(Drug\right)}_{dialysis}\;}{{\nu \left(Drug\right)}_{total}}\times 100\%,$$

where

$$\nu {\left(Drug\right)}_{total}:\;total\;mole\;of\;drug$$

$${\nu \left(Drug\right)}_{dialysis}:\;mole\;of\;drug\;founded\;in\;the\;outer\;solution\;during\;dialysis$$

### 2.8. Complex Preparation

To obtain the complexes, a solution of mannosylated chitosan (5 mg/mL) was added to the suspension of liposomes (5 mg/mL) in a base–molar ratio of 1:7, as was previously found as suitable for biomedical applications [15]. Complexes were incubated for 15 min at room temperature before the following experiments.

### 2.9. Drug Release Kinetics Study

In order to compare drug release kinetics in normal and inflammatory sites, experiments were carried out in 0.02 M sodium phosphate (pH 7.4) and 0.02 M sodium acetate (pH 5.5) buffer solutions. Studies were conducted within the sink conditions. The liposomal suspension was transferred into a dialysis capsule (molecular weight cut-off 10,000, Serva) and placed on a shaker at 37 °C. The volume of release medium was 10 mL, and the volume of the sample was 1 mL. During 25 h, probes of the external solution of 30 μL were taken and replaced by fresh buffer solution (namely, 10, 20, 30, 40, 50, 60, 75, 80, and 90 min, followed by 2, 3, 4, 5, 6, 7, 8, 16, and 25 h). Samples were analyzed with UV–Vis to evaluate the drug concentration in the external solution. All substances under consideration were soluble in the experimental conditions.

The release curves were processed in the coordinates of 0 (Equation (1)) and 1 (Equation (2)) orders, as well as Higuchi (Equation (3)) and Hixson–Crowell (Equation (4)) coordinates, as is described [42,43] according to the following equations:

(1) $$Q_{t}=Q_{0}-K_{0}\times t\;$$

(2) $$\mathrm{lg}Q_{t}=\mathrm{lg}Q_{0}-\frac{K_{1}\times t}{2.303}\;$$

(3) $$Q_{t}=A\times \sqrt{D\times \left(2C-C_{s}\right)\times C_{s}\times t}\;$$

(4) Q013−Qt13=KHC×t 

where $Q_{0}$—initial amount of drug, $Q_{t}$—amount of released drug, $K_{0}$—kinetic constant of 0 order, $\;K_{1\;}$—kinetic constant of the 1st order, A—square of the sample, D—diffusion coefficient, $C_{s}$—solubility of the drug in the matrix, C—initial concentration of the drug, and $K_{HC}$—kinetic constant of Hixson and Crowell.

### 2.10. In Vivo Studies

Black C57 mice were used for in vivo studies. The animals participating in the experiment had no respiratory system disorders and infectious and inflammatory diseases of soft tissues and internal organs of various localization. The experiment was carried out considering the legal and ethical standards, as well as the norms of international public law: “European Convention for the Protection of Vertebrate Animals Used for Experiments or for Other Scientific Purposes (ETS N 123)”. The ethical review was carried out by the local ethical committee of Sechenov University. Mice were kept under standard vivarium conditions with constant free access to food and water, with a 12-h light day.

The aerosol form of the preparations was administered using a veterinary probe. The probe was carefully inserted into the anesthetized animal directly to the carina of the first tracheal bifurcation. The concentrated solution (0.2 mg of moxifloxacin in liposomal and conventional form, a solution of 50 µL) was purposefully delivered by an airless stream directly to the lungs of the animals. As a control group, moxifloxacin was injected into the tail vein of mice after warming up the vein. Before the administration of the preparations, each mouse was anesthetized with 5% isoflurane at a rate of 3 L/min and fixed by the limbs to the surgical table. Mice were hatched at 15 min, 40 min, 90 min, 240 min, 360 min, 24 h, 48 h, and 72 h after drug administration, 3 animals per group. Euthanasia was carried out by a humane and safe method of removal from the experiment using a CO_2_ gas system (EVTANAIZER-2M, INPREN, Moscow, Russia). The lungs were separated, lyophilized, then homogenized (digital homogenizer HG-15D-Unit).

### 2.11. Lung Tissue Studies

Lung tissue samples were prepared for analysis using a variation of the QuEChERS method as following: 10 mg of dry lung tissue was rigorously vortexed with 100 μL H_2_O; after that, 500 μL of acetonitrile was added, and the solution with lumps was shaken and vortexed extensively. Then, 100 mg of QuEChERS kit salts (MgSO_4_, NaCl, disodium citrate, and sodium citrate) from CNW Technologies (Shanghai, China) were added to precipitate proteins, and the mixture was vortexed again. During this part of the procedure, a slight increase in the temperature of the samples could be observed. After that, all samples were centrifuged for 10 min at 10,000 rpm, and the supernatant was transferred into a clean tube for further analysis via fluorescence spectrometry.

In order to accurately determine the concentration of moxifloxacin in the samples, they were registered in different dilution proportions. It is essential, as the samples might contain fluorescence quenching agents. To minimize this effect, each sample was continuously registered and diluted until the signal began to decline upon dilution. Spectra are considered informative when the lowering of the signal occurs two times upon dilution iterations. Subsequently, the acetonitrile background was subtracted from all spectra. To evaluate the fluorescence signal from moxifloxacin, the Varian Cary fluorimeter was used with λex 295 nm and λem 485 nm.

### 2.12. Lyophilization

Samples were freeze-dried for two days at −60 °C (Edwards 5, BOC Edwards, Atlas Copco Group, Stockholm, Sweden).

### 2.13. Statistical Analysis

All experiments were triplicated, and the results were expressed as the mean value ± standard deviation, SD (*n* = 3). AtteStat 3.04 for Microsoft Excel was used for statistical analysis. Significance was analyzed by the Mann–Whitney test, with *p* ≤ 0.05 considered statistically significant.

## 3. Results and Discussion

### 3.1. Liposomal Formulation Preparation

In this work, we set the task of revealing the role of the lipid composition in the interaction of liposomes with drugs. Therefore, we varied the lipid composition relative to the DPPC matrix. The addition of 10% cholesterol gives the membrane, already gelled at room temperature, greater rigidity [44]. Some (20%) cardiolipin in liposomes provides a pronounced anionic charge [24] and creates potential sites for the electrostatic binding of vesicles to mannosylated chitosan [15].

**Figure 1:**
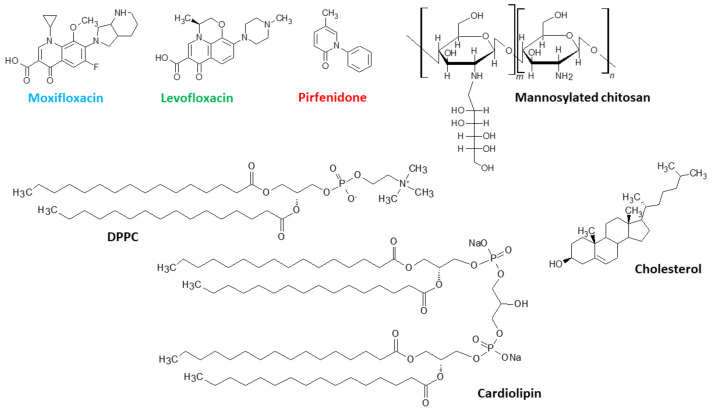
Formulae of the substances under consideration.

We have previously conducted primary studies on the physicochemical properties of liposomal moxifloxacin (DPPC:CL 80:20) [14], levofloxacin (DPPC and DPPC:CL 80:20) [16,45], and pirfenidone (DPPC and DPPC:Chol 90:10) [46]. Here, we aim to conduct a fundamental analysis of the influence of the composition of the lipid matrix on the properties of the liposomal form of these drugs. In what follows, we will refer to the liposomal forms of pirfenidone (Pf), levofloxacin (Lev), and moxifloxacin (Mox) as LPf, LLev, and LMox, respectively. Structures of the all substances under consideration are presented on Figure 1.

Comparing the values of ζ-potentials and the hydrodynamic diameter (Table 1), Dh, we did not find any significant differences in these parameters for the control (“empty”) vesicles and the one loaded with drugs. However, some interesting findings were detected for the encapsulation efficacy.

As it was previously demonstrated [14] for moxifloxacin (Mox), the addition of anionic CL in the lipid matrix composition provides higher EE, while, for DPPC:Chol 90:10, the EE was even lower than for DPPC. Most likely, this may be due to the greater rigidity of the membrane, since moxifloxacin tends to anchor in the internal cavity of the bilayer at the lipid–water interface [14]. However, to confirm this hypothesis, it is advisable to use more informative methods, for example, ATR-FTIR spectroscopy (see Section 3.2).

Levofloxacin is structurally similar to moxifloxacin, since both drugs belong to the class of fluoroquinolones and carry a nalidixic acid moiety in their structure. However, the protonated nitrogen of the moxifloxacin heterocycle does not sterically interfere with the interaction with the anionic groups of lipids. On the contrary, in Lev, the potential site for binding to lipid phosphate groups, the nitrogen of the heterocycle, is methylated, so it can create steric hindrances when interacting with the bilayer. Indeed, no significant EE increase was observed in the experiment. Thus, we could propose that it is the heterocycle that plays a significant role in the interaction with anionic liposomes. Tightening of the DPPC membrane by introducing 10% cholesterol did not lead to a significant change in the efficiency of loading levofloxacin. This correlates well with previous data that levofloxacin does not tend to interact extensively with the lipid-water interface [16,45].

In general, both for Mox and Lev, we have obtained liposomal forms with EE corresponding to the literature [47].

For liposomal forms of pirfenidone, a lower EE was found; only 36 ± 2% was achieved for anionic liposomes. Cardiolipin contributes to a small but statistically significant (*p* < 0.05) increase of EE, indicating a potential interaction between pirfenidone and lipid anionic groups. As it was previously demonstrated [46] even 10% of cholesterol in DPPC matrix leads to a decrease of EE, probably caused by more rigid membrane.

### 3.2. Interaction of Drugs with Lipid Bilayer at Room Temperature

**Figure 2:**
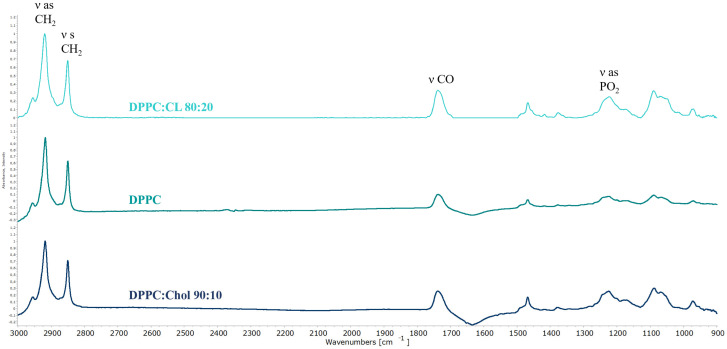
Normalized ATR-FTIR spectra of liposomes of various lipid composition: DPPC:CL 80:20, DPPC, DPPC:Chol 90:10. Total lipid concentration 5 mg/mL. 0.02 M Na phosphate-buffered solution, pH 7.4, 22 °C.

Design and fine tailoring of drug delivery systems involves a thorough study of the mechanism of drug binding to the carrier and the identification of the physicochemical patterns underlying this process. One of the most suitable methods for fine diagnostics of liposomal systems is Fourier IR spectroscopy in the attenuated total reflection regime (ATR-FTIR spectroscopy). This method allows studying heterogeneous colloids [47] and provides detailed information on the state of functional groups of lipids [45,48,49]. When it comes to the analysis of the entire spectral pattern of liposomes there are several absorption bands sensitive to the formation of non-covalent bonds between bilayer and drugs. Figure 2 shows the typical ATR-FTIR spectra of liposomes.

Typical bands of symmetrical (νCH_2_ s) and asymmetric (νCH_2_ as) stretching oscillation of the CH_2_ group are located in the region of 2850 ± 3 cm^−1^ and 2919 ± 6 cm^−1^. These peaks are usually analyzed when it comes to the changes in the mobility of acyl chains [48,49]. The band located in the region of 1715–1750 cm^−1^ corresponds to the valence oscillation of carbonyl groups and can be useful in the analysis of microenvironment on the lipid-water surface. For the phosphate group there are two typical bands of stretching vibrations: νPO_2_^−^ s 1088 cm^−1^ and νPO_2_^−^ as 1250–1230 cm^−1^. Usually in the liposomal studies one analyzes νPO_2_^−^ as because of high sensitivity to the interaction of cationic ligands with the polar head of liposomes. Often for all bands one studies several parameters, e.g., position and shape, presence of shoulders etc. in order to evaluate interaction with different ligands, both polymeric and small molecules [50].

Empty liposomes are in a gel-like state at room temperature, as evidenced by the position of the absorption bands of the acyl chains (Table 2). This is expected based on the composition of the vesicles [51,52]. A slight shift of the νCH_2_ s band for DPPC:CL 80:20 liposomes to the higher wavenumbers potentially indicates a slightly higher mobility of the chains. However, in general, from the point of view of packing of lipids, all three types of liposomes are fairly rigid. On the other hand, from the point of view of the microenvironment of polar lipid groups, anionic liposomes again stand out: we see a certain heterogeneity in the region of absorption of carbonyl groups. This indirectly indicates the existence of two domains in the bilayer [14,53]: rich and depleted in cardiolipin. The presence of the anionic lipid fraction also affects the position of the absorption band of asymmetric stretching vibrations of the phosphate group: 1225 cm^−1^ versus 1223 cm^−1^. It should be noted that for the IR spectra of liposomes, a shift of 2 cm^−1^ is significant, reflecting changes in the microenvironment of this functional group [54].

Previously, we studied the effect of cardiolipin on the nature of the interaction of the DPPC bilayer with moxifloxacin [14] and levofloxacin [16]. For both drugs, the presence of an anionic phospholipid in the composition of the gel-like membrane led to a more intense interaction with liposomes which led to the changes in the absorption area of phosphate and carbonyl groups (Table 2). The difference between Mox and Lev is that if for moxifloxacin there was an unambiguous bright effect of binding to phosphate groups (shift from 1225 cm^−1^ to the 1228 cm^−1^), then for levofloxacin this effect is less pronounced and is accompanied not by a uniform shift of the absorption band, but by the appearance of a shoulder 1265 cm^−1^, characteristic of low hydrated phosphate groups [37]. Thus, for fluoroquinolones, the anionic groups of phospholipids are the key binding sites.

If cholesterol is introduced into the DPPC matrix instead of cardiolipin, the nature of the interaction of moxifloxacin with the bilayer remains the same as for DPPC liposomes. That is, cholesterol does not provide additional binding sites for Mox. For Lev, we observed slight perturbations in the area of absorption of phosphate groups, namely, the appearance of 1235 cm^−1^ shoulder, however, these changes were less pronounced than for liposomes with cardiolipin. Probably the reason for these changes is related to the noncovalent interactions of levofloxacin and cholesterol, but this assumption remains to be tested in subsequent experiments (see Section 3.3).

In contrast, for pirfenidone, cholesterol appeared to be a very important addition to the lipid bilayer. Cholesterol changes the way of interaction between carbonyl groups of lipids in DPPC and DPPC:Chol 90:10 vesicles and causes a dramatic redistribution of carbonyl groups onto the degrees of hydration [46].

Thus, according to the studies at the room temperature liposomes seems to be a suitable carrier for both fluoroquinolones and pirfenidone, so it agrees with previously published data concerning using of liposomes for several innovative drug [55] and gene [56] delivery systems.

To obtain additional information on the mechanism of interaction of active molecules with a bilayer, it is necessary to study the phase transition of the membrane from the liquid-crystal state to the gel state. This process is key to understanding the functioning of liposomal delivery systems, since it is during the phase transition that the most active drug release occurs [52]. ATR-FTIR spectroscopy was chosen as the main research method, since the absorption band of νCH_2_ as vibrations is sensitive to changes in the mobility of hydrophobic chains [54,57].

### 3.3. Influence of the Fluoroquinolones Loading on the Liposomal Phase Transition

The phase transition of gel-like liposomes to the liquid-crystalline phase is accompanied by an increase in the mobility of hydrophobic chains [24]. In the ATR-FTIR spectrum, this is displayed by a shift in the absorption bands of the methylene components to the region of higher wavenumbers [48,54,57]. For gel-like bilayer νCH_2_ as band is located around 2917–2920 cm^−1^, while for liquid–crystalline membrane this band shifts to the 2925 cm^−1^ and more. ATR-FTIR thermogram provides information about the phase transition that is relevant to classical methods, for example, DSC [24,54]. Moreover, ATR-FTIR spectroscopy makes it possible to obtain data on the behavior of individual domains in a bilayer during a phase transition [16,45].

Let us consider the effect of drug loading on the liposome phase transition process. Previously we have found that for DPPC liposomes of loading of moxifloxacin does not have a significant effect: this confirms the assumption that Mox interacts weakly with the mono-component bilayer [14]. On the contrary, the interaction of Mox with DPPC:CL 80:20 bilayer is accompanied by a phase transition slowing down and an anomalous pattern of curves. Two minima were found that are characteristic of two domains: a low-melting domain rich in cardiolipin and a refractory one with a minimum content of CL.

For levofloxacin, we observed other regularities in thermogram curves. Recently we have found that the phase transition of DPPC liposomes significantly accelerates when loaded with Lev [45]: temperature of phase transition shifts from 42 to 38 °C. We proposed that this effect is related with bilayer disordering leading to the more potential binding sites opened for the interaction. In order to study the molecular details of the interaction of levofloxacin with the bilayer during phase transition, we have studied how the shape and position of the absorption bands of the phosphate and carbonyl groups of LLev change during this process.

No anomalous changes were found for carbonyl groups: as for the control unloaded liposomes, the LLev spectra upon heating show splitting of the band into components corresponding to highly and low hydrated forms, and the proportion of the latter noticeably decreases with increasing temperature. This effect has already been described previously [37] and indicates the no specific interaction of levofloxacin with carbonyl groups.

**Figure 3:**
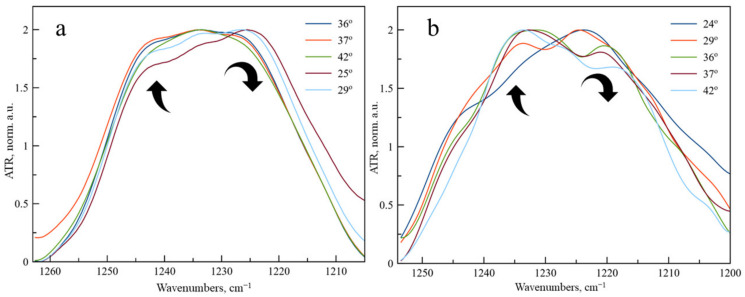
Normalized ATR-FTIR spectra of LLev (**a**) DPPC and (**b**) DPPC:CL 80:20 liposomes at various temperatures. Total lipid concentration 5 mg/mL. 0.02 M Na phosphate-buffered solution, pH 7.4.

On the contrary, for νPO_2_^−^ as the band, we have observed an unusual change in the shape of the band (Figure 3). At temperatures up to 25 °C the main component of νPO_2_^−^ as band is 1222 cm^−1^, corresponding to the unbounded phosphate groups of DPPC exposed to the aqueous media. A shoulder at 1240 cm^−1^ is observed, corresponding to phosphate groups, in the microenvironment of which levofloxacin is located. As the temperature rises, the proportion of the 1222 cm^−1^ component decreases significantly, probably due to the greater availability of phosphate groups and disordering of the bilayer. Such an effect was not previously observed for DPPC drug-loaded liposomes; thus, we were able to detect an unusual character of interaction with the bilayer in Lev.

**Figure 4:**
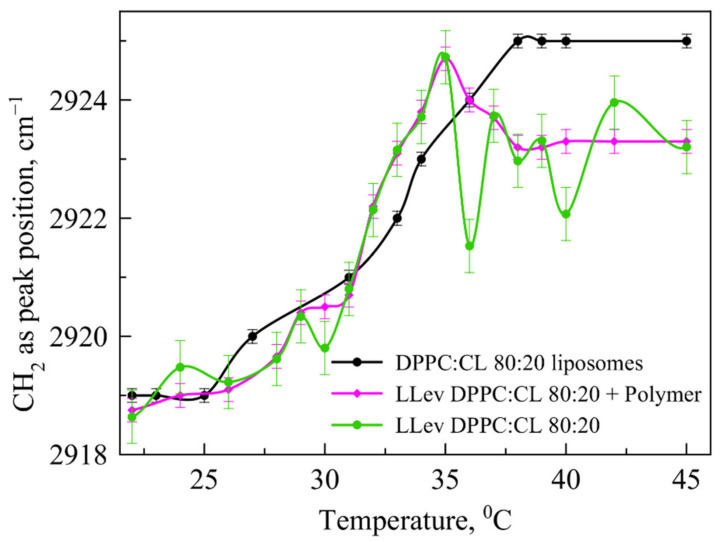
Dependence of νCH2 as the peak position on the temperature for DPPC:CL 80:20 liposomes (black line), LLev DPPC:CL (green line) and complex LLev DPPC:CL with mannosylated chitosan (ChitMan) (magenta line). For each point, the SD (*n* = 3) is presented. Total lipid concentration 5 mg/mL, 0.02 M PBS pH 7.4.

It is well known [14] that the phase transition for anionic liposomes containing 20% of CL (Figure 4, black line) is usually occurs in the range of 35–37 °C, so it requires lower temperature than 100% DPPC vesicles. Recently, we have found [45] that levofloxacin provides more disorder in the membrane with reproducible oscillations in the area of 35–40 °C (Figure 4).

Let us turn again to the changes in the absorption region of the phosphate group (Figure 3b). The significant changes that we observed for DPPC liposomes become even more pronounced for anionic liposomes. With an increase in temperature, the absorption band of the phosphate group completely shifts to the region of large wave numbers: in fact, the softening of the membrane during heating leads to an intense interaction of these functional groups with Lev. This phenomenon has not been previously described in the literature, it is essential for the development of dosage forms of Lev, which should be applied at body temperature.

**Figure 5:**
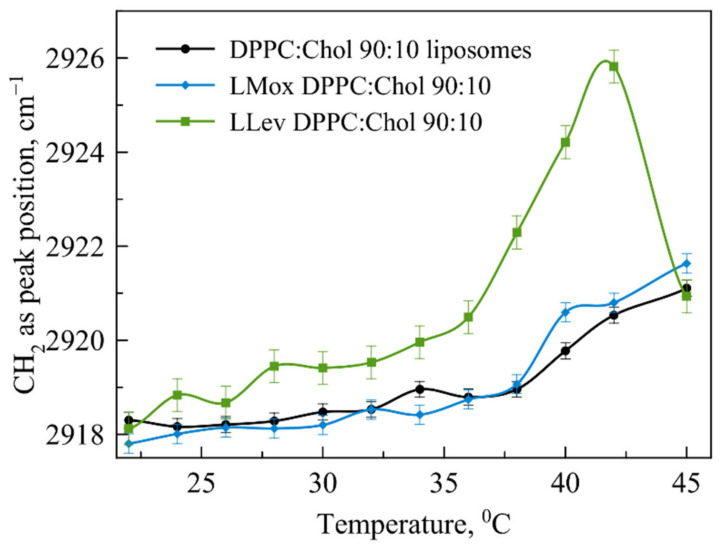
Dependence of wavenumber of νCH_2_as peak position on the temperature for DPPC:Chol 90:10 liposomes (black line), LMox DPPC:Chol 90:10 (blue line) and LLev DPPC:Chol 90:10 (green line). For each point SD is presented (*n* = 3). Total lipid concentration 5 mg/mL. 0.02 M Na phosphate-buffered solution, pH 7.4.

The addition of 10% cholesterol to the DPPC matrix significantly changes the melting pattern of liposomes (Figure 5) loaded with levofloxacin, and practically does not change that for liposomes loaded with Mox. In the absence of an anionic lipid, Mox practically does not affect the phase transition of DPPC:Chol 90:10 liposomes; on the contrary, Lev significantly accelerates it. This acceleration of the phase transition for liposomes loaded with levofloxacin supports the assumption that levofloxacin is able to interact with cholesterol, especially when heated. If at room temperature these effects were weakly noticeable, but when heated to a temperature near the phase transition, this phenomenon manifested itself.

### 3.4. Influence of the Pirfenidone Loading on the Liposomal Phase Transition

**Figure 6:**
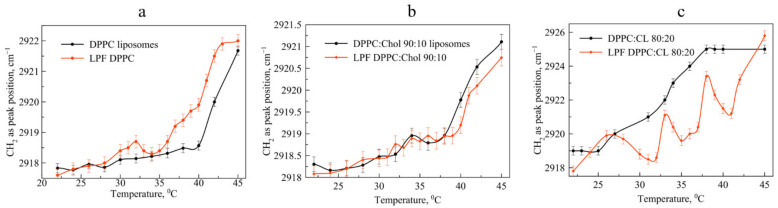
Dependence of wavenumber of νCH_2_as peak position on the temperature for unloaded liposomes (black line everywhere) and pirfenidone-loaded liposomes (red line everywhere). Subfigure (**a**) represents DPPC 100% lipid composition, (**b**) DPPC:Chol 90:10 and (**c**) DPPC:CL 80:20. For each point SD is presented (*n* = 3). Total lipid concentration 5 mg/mL. 0.02 M Na phosphate-buffered solution, pH 7.4.

We observed another pattern for the liposomal forms of pirfenidone. The drug significantly accelerates the phase transition of mono-component liposomes (Figure 6a). At the same time, the addition of cholesterol to the bilayer leads to a disappearance of the effect observed for the DPPC of the bilayer (Figure 6b); thermogram curves have a symbatic course. Thus, the addition of cholesterol makes it possible to ensure the preservation of the physicochemical properties of the bilayer during the transition to temperatures above 30 °C.

An absolutely unexpected result was obtained for the phase transition of LPf DPPC:CL 80:20 anionic liposomes loaded with pirfenidone (Figure 6c). Several areas of minima in the thermogram indicate significant rearrangements in the bilayer in the presence of an active molecule during the phase transition. We have already observed such type of the curve for moxifloxacin, the most lipophilic fluoroquinolone (logP is 2.9 according to the DrugBank). On the other hand, logP values for Pf and Lev are very close to each other: 1.91 and 2.1 correspondingly.

**Figure 7:**
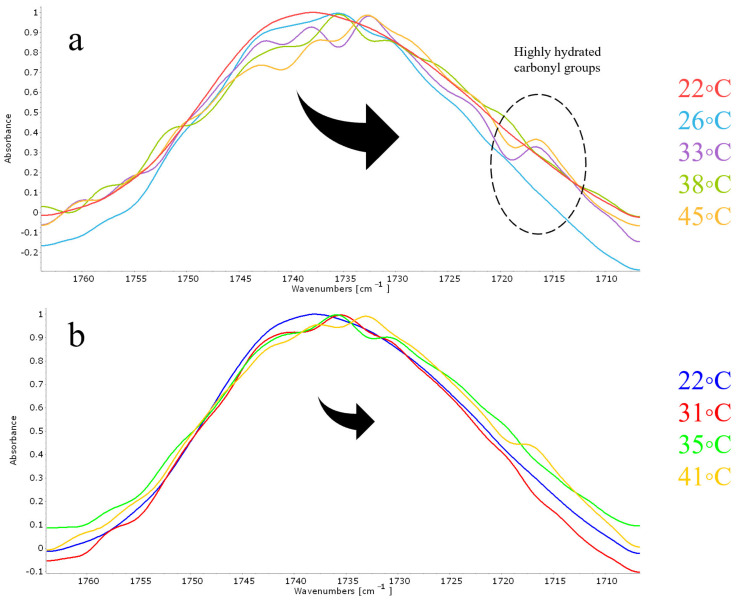
Normalized ATR-FTIR spectra of DPPC:CL 80:20 loaded with pirfenidone: carbonyl group area. (**a**) Spectra recorded at the temperature of maxima on the thermogram. (**b**) Spectra recorded at the temperature of minima on the thermogram. Total lipid concentration 5 mg/mL. 0.02 M Na phosphate-buffered solution, pH 7.4.

In order to understand the nature of such atypical phenomena, we carefully analyzed the absorption region of the carbonyl group at the points on the curve characteristic of the maxima (Figure 7a) and minima (Figure 7b). The minimum on the thermogram indicates a decrease in the mobility of hydrophobic chains, and the maximum, on the contrary, indicates a fluidization of the bilayer. So, the minima of the thermogram (temperatures of 31 °C, 35 °C and 41 °C) are characterized by minimal changes in the region of absorption of the carbonyl group relative to the control spectrum at 22 °C. It is obvious that there are small changes in the microenvironment of carbonyl groups, however, this effect is much weaker than that observed for the points of maxima. For them we observed significant changes in the absorption region of the carbonyl group, primarily associated with the predominance of the components of lower wave numbers characteristic of highly hydrated carbonyl groups. Moreover, at high temperatures, a shoulder of 1717 cm^−1^ appears in the spectrum, which is typical for highly hydrated carbonyl groups. Thus, indeed, during the phase transition of anionic liposomes loaded with pirfenidone, the most severe effect on the bilayer is observed, in which the drug most likely penetrates into the bilayer, provoking the “freezing” of hydrophobic chains. Interestingly, since no such effect on the phase transition was found for either DPPC or DPPC:Chol 90:10 liposomes, cardiolipin plays a critical role in this process. Thus, for the first time at the molecular level, it was shown how, depending on the lipid composition and nature of the drug, the nature of the phase transition of liposomes can be fundamentally changed.

Summarizing this section on the mechanisms of interaction of drugs with liposomes of various compositions, we would like to note that moxifloxacin interacts weakly with DPPC and DPPC:Chol 90:10 vesicles, binding to the surface of the bilayer, mainly due to the formation of non-covalent bonds with the carbonyl and partially phosphate groups of lipids. The incorporation of Mox into anionic liposomes leads to a strong electrostatic interaction with the bilayer and significantly changes the phase transition of liposomes. Levofloxacin has a weak effect on bilayers of various compositions at room temperature, however, during the phase transition, Lev accelerates this process and leads to rearrangements in the absorption region of phosphate groups, especially for anionic liposomes. Pirfenidone interacts with neutral and cholesterol-containing liposomes weakly, due to binding to anionic groups of lipids, and Chol provides more binding sites according to the ATR-FTIR data. A more intense interaction is observed for the system pirfenidone—DPPC:CL liposomes. During the phase transition, the main differences in the interaction of Pf with liposomes appear: DPPC:Chol are not sensitive to the action of the drug, while the phase transition of mono-component liposomes is accelerated. For anionic liposomes, it was possible to detect an atypical course of the curve with bright effects associated with the penetration of Pf into the thickness of the bilayer.

However, the stability of these liposomal forms in suspension during storage does not exceed a week. According to the Deryagin-Landau-Verwey-Overbeck (DLVO) theory, liposomes quickly fuse, aggregate, and lose their homogeneity [58]. In the routine experiments liposomal formulations under consideration were stable not more than 1 week stored in the fridge. Thus, stabilizing additives are needed, for example, based on natural polysaccharide chitosan capable of forming multipoint non-covalent complexes with vesicles [59].

### 3.5. Complex Formation with Mannosylated Chitosan Influences Significantly on the Physico-Chemical Properies of Drug-Loaded Liposomes

Preparation of liposomes loaded with the drug and coated with a polymer shell was carried out by incubation of liposomes with a polymer solution as it was described previously for “empty” liposomes and mannosylated chitosan [15]. We have chosen DPPC:CL liposomes as a matrix for Mox and Pf as the most interesting effects were observed for this lipid composition as well as higher values of EE were archived. Moreover, anionic surface provides these liposomes more opportunities for electrostatic interaction with polymer. Unfortunately for Lev DPPC:CL composition was unstable upon phase transition (Figure 4, green line), thus for the following studies we have considered both DPPC and DPPC:CL liposomes as we proposed that complex formation could stabilize bilayer. For DPPC:CL liposomes, the base molar ratio with mannosylated chitosan is 1:7 in terms of phosphate groups of cardiolipin and free amino groups of chitosan. For liposomes of a different composition, the same proportions of the components of the complex are observed.

The mechanism of interaction between mannosylated chitosan and anionic liposomes is electrostatic, as evidenced by the characteristic high-frequency shifts of the absorption bands of the carbonyl and phosphate groups (Table 3). In all cases, there is an increase in the Dh of vesicles by 15–40 nm, depending on the nature of the bilayer, and neutralization of the ζ-potential, which indicates the electrostatic complex formation with following polymer shell. The observed processes in terms of physicochemical regularities corresponded to those previously described by us for unloaded liposomes complexed with other chitosan derivatives [15,37,60].

We believed [37] that LLev DPPC:CL 80:20 complexation with mannosylated chitosan would prevent damage to the bilayer during the phase transition. To check this, in an independent experiment we have obtained a thermogram of such a complex and compared it with those for empty liposomes and LLev without a polymer shell (Figure 4, magenta line). Indeed, in the most significant temperature range corresponding to physiological values, complex formation made it possible to prevent disturbance in the bilayer; the phase transition is completed by 40 °C and the liposomes pass into the liquid crystal state. Thus, even despite the instability of the initial liposomal form of levofloxacin, it was possible to stabilize it due to complex formation.

### 3.6. Drug Release Kinetics Studies

For liposomal formulations, the key parameter is the rate of drug release. We compared the release kinetics from the selected formulations in order to determine the effect of the polymer on this process. Will it be possible to provide a sustained release from the vesicles?

**Figure 8:**
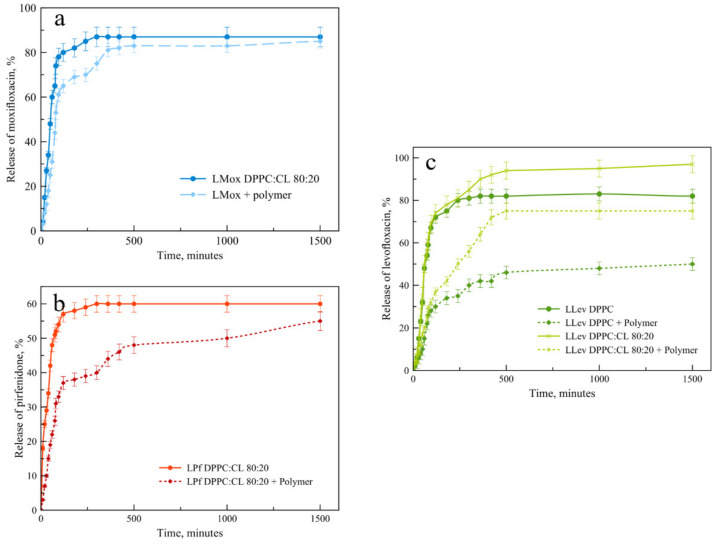
Drug release from liposomal forms of drugs with and without a polymer shell. (**a**) LMox DPPC:CL 80:20 and its complex with mannosylated chitosan, base–molar ratio 1:7. (**b**) LPf DPPC:CL 80:20 and its complex with mannosylated chitosan, base–molar ratio 1:7. (**c**) LLev DPPC and its complex with mannosylated chitosan, base–molar ratio 1:7 and LLev DPPC:CL 80:20 at the same conditions. Total lipid concentration 3 mg/mL in 0.02 M Na phosphate-buffered solution, pH 7.4. 37 °C. SD (*n* = 3), *p* < 0.05.

Considering the course of the drug release curves from liposomal forms and their complexes with mannosylated chitosan (Figure 8), we have proved that the formation of the complex does indeed provide sustained release. Interestingly, this effect is weakest for moxifloxacin, while for pirfenidone, the extended release effect is very pronounced. We will pay special attention to the drug release pattern for the liposomal form of levofloxacin (Figure 8c). Here we compare two compositions of the lipid matrix. Dark green shows plots for DPPC matrix, and light green for DPPC:CL 80:20. Obviously, without the polymer shell of anionic liposomes, the release is faster. This is in good agreement with the previously obtained data on thermograms, since the release itself takes place at a temperature of 37 °C. For both types of lipid compositions, complex formation leads to a significant slowing down the release, and the complex of neutral liposomes with the polymer turned out to be the slowest of all the samples considered. In this case, it was not possible to achieve the release of even half of the loaded cargo during the 25 h.

Mathematical processing of the release curves was carried out in zero and first-order coordinates, as well as according to the Higuchi and Hixson–Crowell equations in order to identify the most reliable ones [42]. Table S1 (Supplementary Materials) presents the values of all R^2^; the release kinetics is most reliably described by zero-order equations with a mean R^2^ 0.958 and SD 0.023, which corresponds to the literature data for liposomal forms of antibiotics [43]. Figure S1 presents all release curves’ linearization in the zero-order coordinates.

**Figure 9:**
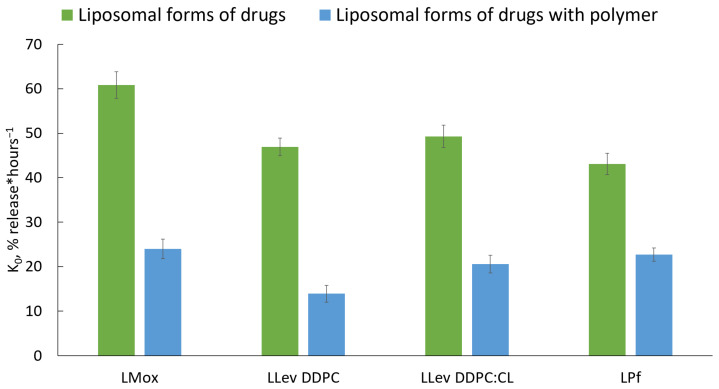
Zero-order kinetic constants of drug release from liposomal forms with (blue) and without (green) a polymer shell. Liposomal formulations: LMox DPPC:CL 80:20, LLev DPPC, LLev DPPC:CL 80:20, and LPf DPPC:CL 80:20 and its complexes with mannosylated chitosan, base–molar ratio 1:7. Total lipid concentration was 3 mg/mL in 0.02 M Na phosphate-buffered solution, pH 7.4. 37 °C. SD (*n* = 3), *p* < 0.05.

Comparing constants of release k_0_ (Figure 9), we definitely conclude that complex formation provides ca. two to three times slower drug release. The most significant change was observed for LLev DPPC 100% vesicles: k_0_ decreases more than three times. These results are in a good agreement with previously published data concerning polymeric shells. Thus, a complex formation could be considered a perspective technique, as it provides sustained release in the neutral medium, which is desired for fluoroquinolones and pirfenidone.

### 3.7. In Vivo Studies of Biodistribution of Drugs in Mice

To assess the therapeutic effect of accumulation in lung tissues, a sample of the liposomal form of moxifloxacin in combination with mannosylated chitosan was chosen, since it is characterized by a fairly rapid release, which is convenient for in vivo pilot trials.

**Figure 10:**
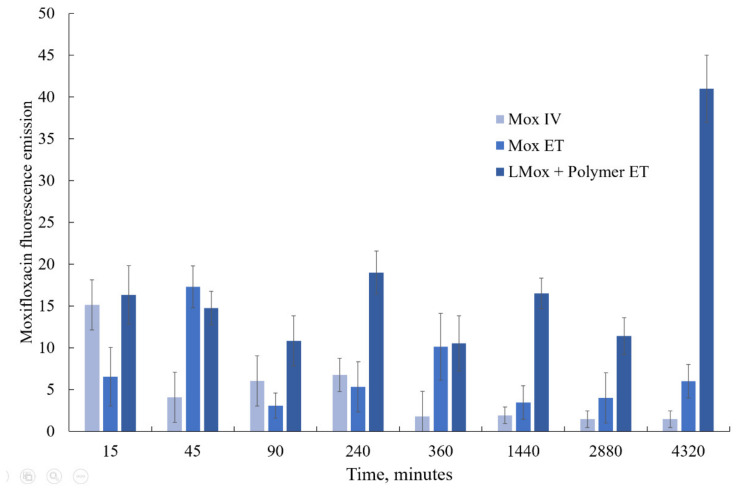
Accumulation of moxifloxacin in the lungs of mice after intravenous (IV) and endotracheal (ET) administration according to the fluorescence emission data. SD (*n* = 3), *p* < 0.05.

We studied the accumulation of moxifloxacin in lung tissues (Figure 10) after the administration of the drug itself and its liposomal form LMox DPPC:CL 80:20 + Mannosylated chitosan endotracheally. As a control, we injected moxifloxacin into the tail vein.

With the intravenous administration of moxifloxacin, there is a sharp surge in its content in the lungs after 15 min and the same rapid elimination. The use of endotracheal administration contributes to a longer stay of the drug in the lungs; however, after a day, the drug is almost completely removed from the tissue. A different picture is observed for the liposomal formulation of moxifloxacin decorated with mannosylated chitosan. Here, during the first day, a sufficiently high level of the drug content is maintained, and then, after 72 h, the concentration of moxifloxacin already increases. This seems to be due to the slow release of the drug and the increased affinity of liposomes and chitosan for lung tissue.

## 4. Conclusions

Delivery systems for inhaled antifibrotics and antibacterial drugs may prove most useful in treating the severe effects of COVID-19. In this work, we attempted to create liquid–polymer delivery systems based on liposomes loaded with pirfenidone, moxifloxacin, and levofloxacin. As it turned out, it was possible to control the physicochemical processes of drug binding to the bilayer using the lipid composition of vesicles. However, not all patterns found at room temperature are retained upon transition to physiological processes, as evidenced by the key rearrangements in the bilayer during the phase transition in the presence of drugs. The functionalization of liposomal forms with mannosylated chitosan made it possible to significantly slow down the release of the contents, as evidenced by the processing of the release curves.

This approach justified itself during in vivo testing. Compared with intravenous and endotracheal administration of free moxifloxacin, the liposomal drug accumulates much more in the lungs and is released slowly: a day after administration, the drug is still present in significant amounts in the tissues, while the control systems are rapidly eliminated.

This result makes it possible to plan subsequent experiments that will allow the creation of inhaled forms of sustained-release drugs, which will make it possible to build the most effective therapy for infectious diseases.

## Figures and Tables

**Table 1:** Encapsulation efficacy, ζ-potential and hydrodynamic diameter of liposomal form of moxifloxacin, levofloxacin and pirfenidone of various lipid compositions; 0.02 M Na phosphate-buffered solution, pH 7.4, 22 °C.

Drug	Lipid Composition	Encapsulation Efficacy, %	ζ-Potential, mV	Dh, nm
Empty liposomes	DPPC		−10.1 ± 0.5	103 ± 2
	DPPC:Chol 90:10		−12.5 ± 0.7	100 ± 3
	DPPC:CL 80:20		−20.0 ± 1.0	100 ± 2
Moxifloxacin	DPPC	66 ± 3	−10.7 ± 0.5	99 ± 1
	DPPC:Chol 90:10	62 ± 4	−13.0 ± 0.4	104 ± 2
	DPPC:CL 80:20	71 ± 2	−20.2 ± 0.6	100 ± 3
Levofloxacin	DPPC	55 ± 2	−10.3 ± 0.2	100 ± 2
	DPPC:Chol 90:10	55 ± 4	−13.0 ± 0.5	97 ± 3
	DPPC:CL 80:20	51 ± 7	−21.5 ± 1.0	103 ± 3
Pirfenidone	DPPC	30 ± 3	−13.1 ± 0.6	107 ± 6
	DPPC:Chol 90:10	20 ± 3	−10.9 ± 0.5	98 ± 2
	DPPC:CL 80:20	36 ± 2	−20.1 ± 0.5	106 ± 4

**Table 2:** Main band position on the ATR-FTIR spectra of liposomal forms of drugs. Total lipid concentration 5 mg/mL. 0.02 M Na phosphate-buffered solution, pH 7.4, 22 °C. Spectral resolution 1 cm^−1^. Shoulders of bands are marked with “sh”.

Drug	Lipid Composition	νCH_2_ as, cm^−1^	νCH_2_ s, cm^−1^	νCO, cm^−1^	νPO_2_^−^ as, cm^−1^
Empty liposomes	DPPC	2918	2850	1737	1223
	DPPC:Chol 90:10	2918	2850	1738	1224
	DPPC:CL 80:20	2918	2851	1739, 1723 sh	1225
Moxifloxacin	DPPC	2918	2850	1737	1223
	DPPC:Chol 90:10	2918	2850	1737	1224
	DPPC:CL 80:20	2918	2851	1742	1228
Levofloxacin	DPPC	2919	2850	1740, 1725 sh	1221, 1240
	DPPC:Chol 90:10	2919	2850	1738	1223, 1235 sh
	DPPC:CL 80:20	2918	2851	1740	1220, 1225, 1265 sh
Pirfenidone	DPPC	2918	2850	1738	1223, 1243 sh
	DPPC:Chol 90:10	2918	2850	1736	1224, 1243 sh
	DPPC:CL 80:20	2918	2850	1738	1223, 1243 sh

**Table 3:** Main band position on the ATR-FTIR spectra of liposomal forms of drugs and its complexes with mannosylated chitosan. Total lipid concentration 3 mg/mL. 0.02 M Na phosphate-buffered solution, pH 7.4, 22 °C. Spectral resolution 1 cm^−1^. Shoulders of bands are marked with “sh”. For DPPC:CL 80:20 liposomes, the base molar ratio with Mannosylated chitosan is 1:7 in terms of phosphate groups of cardiolipin and free amino groups of chitosan. For liposomes of a different composition, the same proportions of the components of the complex are used.

Drug	Lipid Composition	Sample	νCH_2_ as, cm^−1^	νCH_2_ s, cm^−1^	νCO, cm^−1^	νPO_2_^−^ as, cm^−1^	ζ-Potential, mV	Dh, nm
Moxifloxacin	DPPC:CL 80:20	LMox	2918	2851	1742	1228	−20.2 ± 0.6	100 ± 3
		LMox + polymer	2918	2850	1740	1225, 1243, 1263 sh	+6 ± 3	116 ± 2
Levofloxacin	DPPC	LLev	2919	2850	1740, 1725	1221, 1240 sh	−10.3 ± 0.2	100 ± 2
		LLev + polymer	2919	2850	1740, 1730	1225, 1243 sh	+7 ± 2	116 ± 3
	DPPC:CL 80:20	LLev	2918	2851	1740	1220, 1225, 1265	−21.5 ± 1.0	103 ± 3
		LLev + polymer	2917	2850	1737	1235	+6 ± 4	128 ± 8
Pirfenidone	DPPC:CL 80:20	LPf	2918	2850	1740	1223, 1243 sh	−20.1 ± 0.5	106 ± 4
		LPf + polymer	2918	2850	1740	1223, 1243 sh	+10 ± 2	118 ± 3

## Data Availability

Not applicable.

## Supplementary Materials

The following supporting information can be downloaded at: https://www.mdpi.com/article/10.3390/pharmaceutics15041101/s1, Table S1. Values of R^2^ for release kinetics approximations in different linearization models; Figure S1. Linearization of release curves for liposomal forms of Mox, Lev and Pf. Liposomal formulations: LMox DPPC:CL 80:20, LLev DPPC, LLev DPPC:CL 80:20, LPf DPPC:CL 80:20 and its complexes with mannosylated chitosan, basemolar ratio 1:7. Total lipid concentration 3 mg/mL. 0.02 M Na-phosphate buffer solution, pH 7.4. 37 °C. SD (*n* = 3), *p* < 0.05.
