# Supplementary material for: Liposomal Forms of Fluoroquinolones and Antifibrotics Decorated with Mannosylated Chitosan for Inhalation Drug Delivery
Source: Pharmaceutics. 2023 Mar 29;15(4):1101. doi: 10.3390/pharmaceutics15041101 (PMC10145208; doi:10.3390/pharmaceutics15041101)
Supplement: Supplementary file 1 [file pharmaceutics-15-01101-s001.zip › pharmaceutics-2283901-supplementary.pdf]

Supplementary materials for the manuscript

"Liposomal Forms of Fluoroquinolones and Antifibrotics Decorated with Mannosylated Chitosan for Inhalation Drug Delivery"

Table S1. Values of R^2 for release kinetics approximations in different linearization models.

Sample	0 order	1 order	Hixon-Crowell	Higuchi
LMox	0,971	0,938	0,952	0,803
LMox + Polymer	0,930	0,911	0,918	0,753
LLev DPPC	0,940	0,924	0,910	0,749
LLev DPPC + Polymer	0,969	0,960	0,964	0,838
LLev DPPC:CL	0,940	0,884	0,901	0,736
LLev DPPC:CL + Polymer	0,977	0,970	0,973	0,833
LPf	0,946	0,974	0,967	0,984
LPf + Polymer	0,996	0,994	0,995	0,890
Mean value	0,958	0,944	0,947	0,823
SD	0,023	0,037	0,034	0,084

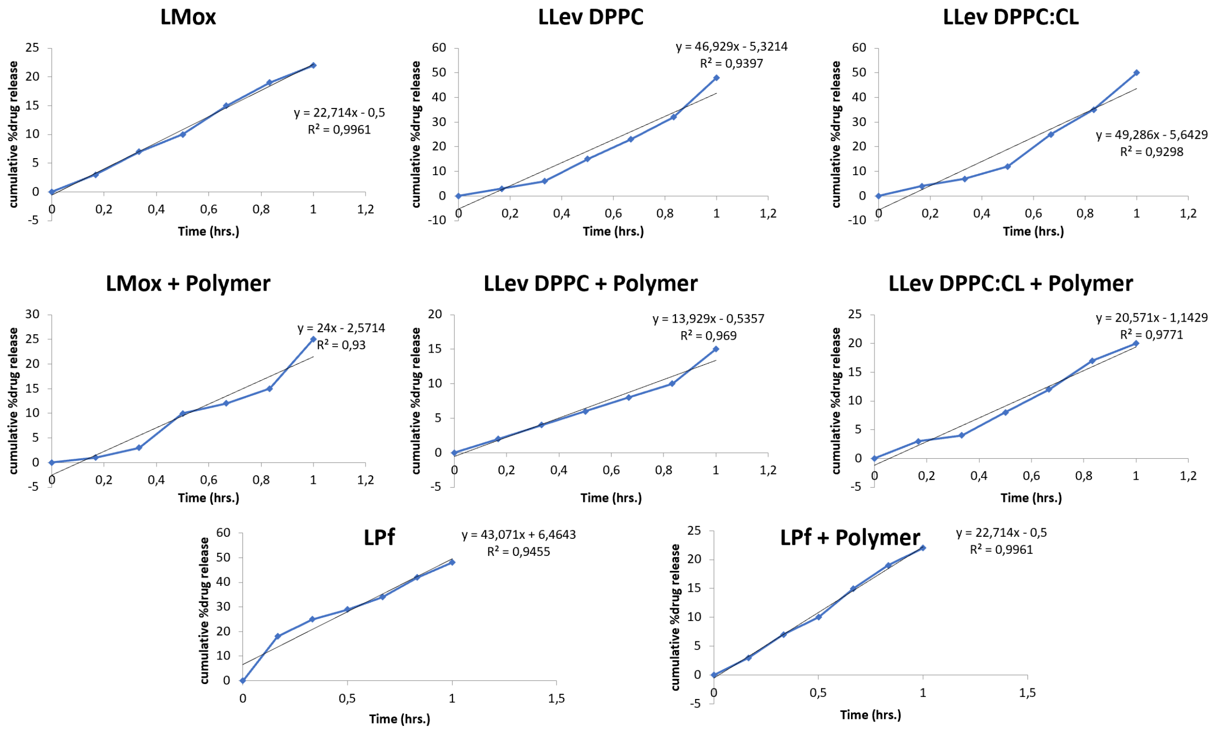

Figure S1. Linearization of release curves for liposomal forms of Mox, Lev and Pf. Liposomal formulations: LMox DPPC:CL 80:20, LLev DPPC, LLev DPPC:CL 80:20, LPf DPPC:CL 80:20 and its complexes with mannosylated chitosan, basemolar ratio 1:7. Total lipid concentration 3 mg/mL. 0,02 M Na-phosphate buffer solution, pH 7.4. 37 °C. SD (n=3), $p<0.05$.
